# Impact of air‐polishing with erythritol on exposed root dentin: A randomized clinical trial

**DOI:** 10.1111/idh.12835

**Published:** 2024-06-09

**Authors:** Anne Brigitte Kruse, Ann‐Sophie Burkhardt, Kirstin Vach, Elmar Hellwig, Johan Peter Woelber, Nadine Schlueter, Petra Ratka‐Krüger

**Affiliations:** ^1^ Department of Operative Dentistry and Periodontology, Faculty of Medicine University of Freiburg Freiburg Germany; ^2^ Department of Medical Biometry and Statistics, Faculty of Medicine University of Freiburg Freiburg Germany; ^3^ Policlinic of Operative Dentistry, Periodontology, and Pediatric Dentistry, Medical Faculty Carl Gustav Carus Technische Universität Dresden Dresden Germany; ^4^ Hannover Medical School, Department of Conservative Dentistry Periodontology and Preventive Dentistry Hannover Germany

**Keywords:** professional mechanical plaque removal, profilometry, rubber cup polishing, surface roughness

## Abstract

**Introduction:**

The effects of air‐polishing on exposed root dentin surfaces are largely unknown, as there are only few studies which show heterogeneous results. Thus, this study was to investigate roughness changes of exposed dentin surfaces after air‐polishing and the influence of subsequent polishing with cup and paste.

**Methods:**

Totally 54 teeth with exposed root dentin surfaces were treated using a split‐mouth design by either air‐polishing with erythritol and a rubber cup with polishing paste on the test side, or rubber cup and paste alone. Teeth were finally cleaned using a sonic tooth brush. Impressions were taken at relevant time points and replicated using epoxy resin. The resulting casts were profilometrically analysed to obtain the average surface roughness (s*R*_a_) and maximum peak‐to‐valley height (s*R*_z_), which are given as the mean ± standard deviation in μm.

**Results:**

After air‐polishing, in comparison to the baseline, there was a slight but significant increase in s*R*_a_ (0.168 ± 0.143, *p* < 0.001), but s*R*_z_ did not change (−0.471 ± 4.857, *p* = 0.936). Subsequent polishing with cup and paste and cleaning with a sonic toothbrush did not reduce the surface roughness (sonic toothbrush‐air‐polishing, s*R*_a_ −0.044 ± 0.081, *p* = 0.218; s*R*_z_ −0.551 ± 3.563, *p* = 0.903).

**Conclusion:**

The use of erythritol led to a slight increase in the roughness of the dentin surface, which was not reduced by polishing with a cup and paste. Polishing paste did not seem to conceal surface irregularities.

## 1 INTRODUCTION

Dental biofilm is considered the main etiologic factor in the development of gingivitis and periodontitis.^1^ Therefore, in addition to reducing established risk factors such as smoking and diabetes,^2^ biofilm removal is an essential part of prevention and treatment strategies for these diseases. The removal of supragingival biofilm from enamel and exposed dentin surfaces during a dental session, also referred to as professional mechanical plaque removal (PMPR), is usually achieved using either air‐polishing devices, rotating rubber cups or polishing brushes in combination with polishing pastes. Highly abrasive powder substrates such as sodium bicarbonate are only recommended for air‐polishing of supragingival aspects on intact enamel,^3^, ^4^, ^5^ and low‐abrasive powders such as glycine, erythritol or trehalose have been described as suitable for use on exposed root dentin.^5^, ^6^, ^7^, ^8^ Several studies have proven the safety of these low‐abrasive powders with regard to soft tissue.^5^, ^9^, ^10^ However, it remains largely unclear what effects these procedures have on surface roughness parameters, especially for exposed root dentin surfaces. While several older studies have investigated the use of air‐polishing with sodium bicarbonate powder,^3^, ^5^, ^11^ only a few studies using low‐abrasive powder substrates are available, and these studies have high heterogeneity in terms of the study parameters such as the surface investigated, the powder substrate used, the application time, and the distance from the surface.^5^, ^12^, ^13^, ^14^, ^15^ An in vitro study comparing different combinations of the use of air‐polishing with erythritol, rubber cup and paste, ultrasonic instrumentation and hand instrumentation showed that air polishing on root cementum led to the lowest loss of substance, but at the same time to the highest maximum peak‐to‐valley height (*R*_z_).^12^ Another in vitro study investigated the defect depth for dentin after the use of erythritol, comparing different working angles and distances under constant spraying. An average surface roughness (*R*_a_) of 0.352 μm was found, compared to 0.030 μm before the treatment.^13^ A further study used scanning electron microscopy to investigate the effect of erythritol and polishing paste on enamel.^16^ The study found that air polishing with erythritol was more likely to expose and visualize existing surface finenesses due to deep cleaning, while polishing with cup and paste flattened the surface. It was suggested that the paste might smear these areas and thus conceal the surface morphology. In a recently published ex‐vivo study by this working group the effects on roughness of pellicle‐coated dentin samples by using a curette, air polishing, polishing with cup and paste as well as combinations of these procedures were examined.^17^ Furthermore, after the different procedures the samples were cleaned from all residues by using an ultrasonic bath. Here a mean significant increase of 0.16 μm for surface roughness (s*R*_a_) was found for the use of air polishing on natural dentin surfaces. The use of RDA 40 polishing paste and a rubber cup led to no significant increase of s*R*_a_ nor s*R*_z_.^17^

To the authors' knowledge, no clinical study has yet investigated the influence of air‐polishing with low abrasive powders on dentin roughness. Therefore and also to verify the results of the ex vivo study, the aim of this clinical trial was to investigate the effect of air‐polishing with erythritol on the average surface roughness (s*R*_a_) and the highest maximum peak‐to‐valley height (s*R*_z_) of exposed dentin and the influence of a subsequent polishing step using a rubber cup and paste. Whether the paste was retained on the surface, thereby concealing the surface morphology was also investigated. For this, any remaining polishing paste residue was removed from the dentin surface using a sonic toothbrush.

## 2 MATERIALS AND METHODS

### 2.1 Ethics approval and informed consent statement

This clinical trial was conducted in accordance with good clinical practice guidelines and respected the principles of the Declaration of Helsinki related to human experimentation.^18^ The study protocol was approved by the Ethics Committee of the University Medical Center Freiburg with a positive vote granted on 3/12/2020 (EK No. 625/19). All enrolled participants gave their written informed consent for study participation and signed a data privacy statement. This report follows the criteria of the CONSORT statement.^19^ Before the start of recruitment, the study was registered in an international trial register (German Clinical Trial Register number DRKS00021624, registration date 5/11/2020, https://drks.de/search/de/trial/DRKS00021624).

### 2.2 Study design

A split‐mouth design was used in this randomized controlled trial. Two nonadjacent teeth from 30 participants were chosen and randomly assigned to either the test or control group. The primary endpoint was the change in roughness parameters s*R*_a_ and s*R*_z_ after treatment with air‐polishing in comparison to the baseline. Secondary endpoints were the change in roughness after subsequent polishing with a rubber cup and polishing paste and after a final step of cleaning using a sonic toothbrush.

### 2.3 Recruitment

**FIGURE 1:**
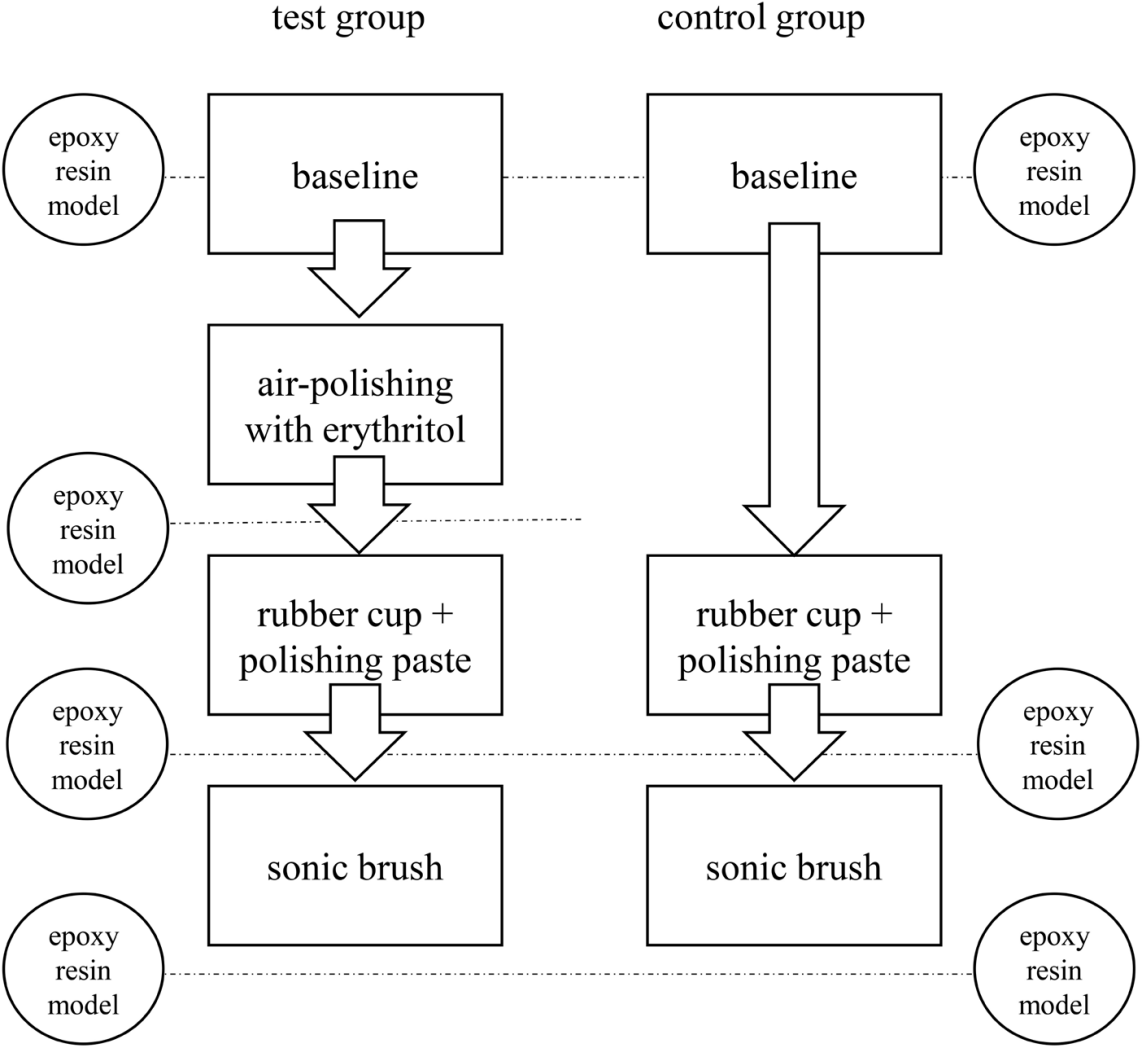
Consort flow diagram.

The participants were recruited from patients at the Department of Operative Dentistry and Periodontology, Faculty of Medicine and Medical Center, University of Freiburg, Germany. Recruitment took place between August 2020 and August 2021 (Figure 1 Consort flow diagram).

### 2.4 Inclusion and exclusion criteria

The inclusion of participants was based on the following criteria: two nonadjacent teeth with vestibular gingival recessions of at least 3 mm depth and a maximum periodontal pocket depth of 4 mm without bleeding on probing. The teeth had to be incisors, canines or premolars. The exclusion criteria were caries, noncarious cervical lesions or hypersensitivity of the designated teeth, asthma, chronic bronchitis, infection of the upper respiratory tract, immunosuppression, increased risk of endocarditis, allergies to erythritol, silica or chlorhexidine, and pregnancy.

### 2.5 Clinical examination and preparation

For both designated teeth, the recession width, recession depth and periodontal pocket depths were recorded. The vestibular surfaces of the study teeth were then stained with a plaque revelator to facilitate the removal of dental biofilm using a Gracey curette No. 7/8 (Hu‐Friedy, Frankfurt a.M., Germany). Subsequently, the tooth surfaces were wiped with a foam pellet soaked in sodium hypochlorite to remove plaque remnants. For a better view and easier suction, the lips were held away with a special holder (Optragate, Ivoclar Vivadent, Ellwangen, Germany). All examinations and treatment steps were carried out by the same trained investigator (dentist, ASB) at a single appointment for each participant at the Department of Operative Dentistry and Periodontology, Faculty of Medicine and Medical Center, University of Freiburg, Germany.

### 2.6 Randomization

In the course of randomization, it was determined which tooth (1 or 2) should be treated with the respective method (A or B). Teeth were numbered from distal of the first quadrant to distal of the second quadrant and then from distal of the third quadrant to distal of the fourth quadrant in ascending order. Treatment A was defined as air‐polishing with erythritol (test group), and treatment B was defined as polishing with cup and paste alone (control group). A randomization list was compiled by a statistician (KV) using STATA software (StataCorp LT, College Station, TX, USA, version 17.0).

### 2.7 Interventions in the experimental and control groups

**FIGURE 2:**
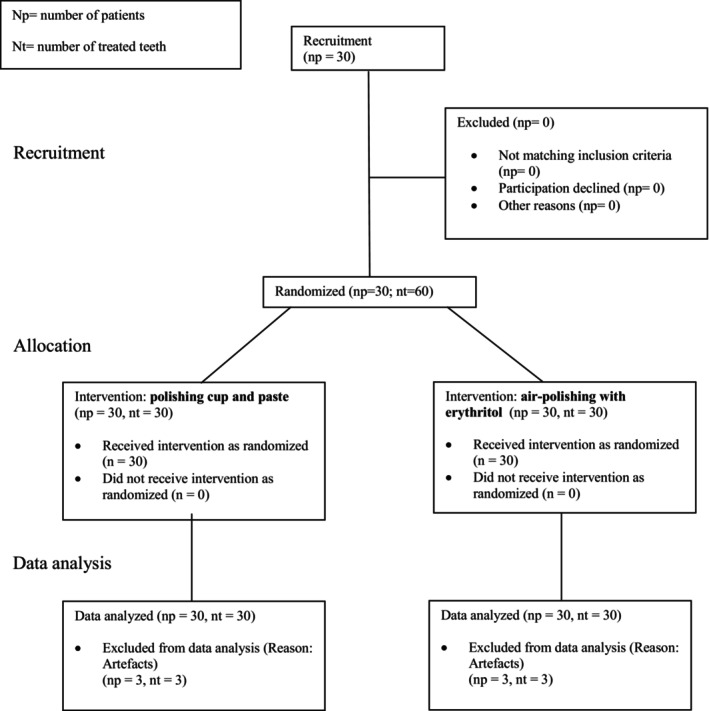
Study workflow.

The experimental group was treated in the cervical area of exposed dentin with erythritol powder with a mean particle size of 14 μm for 5 s under constant movement (Air‐Flow PLUS powder, EMS Dental, Nyon, Switzerland) (step I, test group only). Afterward, polishing with a commonly used rubber cup (rubber cup blue Wkst205.3, Alfred Becht, Offenburg, Germany) and polishing paste (RDA 40; ProphyCare® Prophy Paste CCS, Directa, Upplands Väsby, Sweden) was performed for 5 s under moderate pressure (approximately 150 g) and circular movement (step II, both groups). To remove any polishing paste residue, the surface was cleaned using a sonic toothbrush for 5 s (Sonicare 4300 Protective Clean, Philips, Hamburg, Germany) as a final procedure (step III, both groups). After each step, as well as at the baseline, a partial jaw impression was performed and used to make epoxy resin casts.^20^ Figure 2 shows the workflow for the test and control groups.

### 2.8 Replica technique and profilometrical analysis

At the baseline and after each intervention, partial jaw impressions were obtained using low viscosity silicone (Aquasil Ultra+ regular and extra‐low viscosity, Dentsply Sirona, Charlotte, USA). A total of seven impressions were produced during the study session: after biofilm removal (baseline, control and test groups), after air‐polishing (test group only), after polishing with a rubber cup and paste (control and test groups) and after cleaning with a sonic toothbrush (control and test groups, Figure 2). From the impressions of the treated teeth, highly precise epoxy resin casts (Biresin G30, Sika AG, Baar, Switzerland) were made. These casts were scanned with an optical profilometer using a chromatic‐confocal point topography sensor (vertical measuring range 300 μm, MikroProf 100, Acquire, FRT GmbH, Bergisch Gladbach, Germany). The measuring software Mark III was used to analyse the 3D scans (FRT GmbH, Bergisch Gladbach, Germany) to obtain the arithmetical average roughness (s*R*_a_) and maximum peak‐to‐valley height (s*R*_z_) values (given in μm). The measuring field was located in the area of the exposed and treated dentin and was 1 mm (*x*‐axis) × 1 mm (*y*‐axis) in size. In addition, 3D visualizations of scans of representative surfaces were additionally constructed from randomly selected casts.

### 2.9 Precision of the measuring method

For analysis of the precision of the method, impressions were taken of five extracted teeth, from which replica were made. From both, the natural teeth and the replica the roughness‐values s*R*_a_ and s*R*_z_ were measured. A Bland–Altman analysis of the measurements showed for s*R*_a_ a linear equation of *y* = 0.0924*x* – 0.0074 and for s*R*_z_ of *y* = −0.0087*x* + 2.6506. For both parameters, the slope was nearly zero, meaning that there was no relative bias. For s*R*_a_ no proportional bias was found, as the intercept was also nearly zero. For s*R*_z_, a small proportional bias was found; the replica slightly overestimated the roughness. The reproducibility of the whole procedure including the impression and replica technique as well as the positioning of the replica in the measurement system was measured by taking five impression from one tooth and making five replica from it. The mean and standard deviation for such five processes was for s*R*_a_ 0.362 ± 0.032 and for s*R*_z_ 5.318 ± 0.826, showing low SD values compared to the respective mean value.

### 2.10 Sample size calculation

In a dissertation from the University of Giessen,^21^ changes in roughness (s*R*_a_) between 0.013 and 0.381 μm were observed after treating prepared root surfaces (dentin, plane specimens, human) with air‐polishing (various powders, glycine and erythritol). To demonstrate clinically relevant group differences of 0.15 μm with a power of 0.9 and an alpha of 0.05, 30 teeth per group were needed, taking into account expected dropouts.

### 2.11 Statistical analyses

For the descriptive analysis, the mean values and standard deviations were calculated, and boxplots, scatter plots and line plots were created for graphical representation. Linear mixed models were used to compare the roughness values within and between the groups, and Scheffe's method was used to adjust for multiple testing. A paired *t*‐test was carried out for individual comparisons. All analyses were performed with the statistical software STATA (StataCorp LT, College Station, TX, USA, version 17.0). The level of statistical significance was set at 0.05.

## 3 RESULTS

### 3.1 Recruitment and demographic data

A total of 30 participants meeting the inclusion criteria were recruited and enrolled in the study. Data from these 30 participants were preliminarily analysed, but 27 were included in the final analysis due to artefacts (Figure 1 Consort flow diagram). Eleven of these participants were female and 16 were male. Fifteen per cent reported regular tobacco use. The mean age was 57.4 ± 10.6 years, with a range of 26–72 years.

### 3.2 Surface roughness

#### 3.2.1 s*R*_a_

**FIGURE 3:**
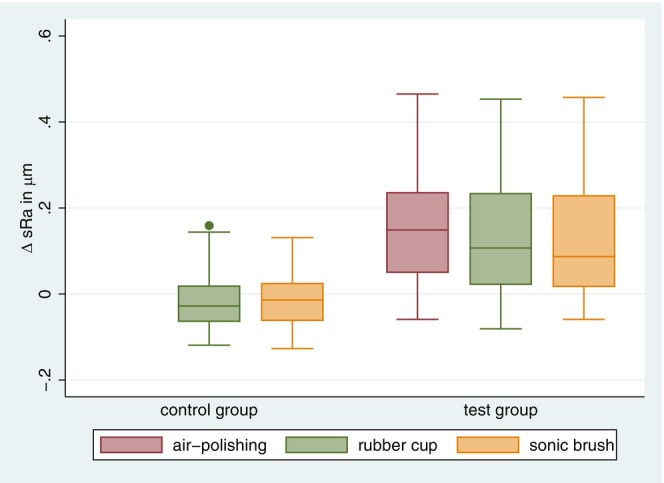
Differences in s*R*_a_. Boxplots showing differences in s*R*_a_ (in μm) between the air‐polishing, rubber cup and sonic toothbrush steps for the test group and between the rubber cup and sonic toothbrush steps for the control group in comparison to the baseline.

After air‐polishing, the test group showed a significant increase in s*R*_a_ with a mean of 0.17 μm (*p* < 0.05), whereas polishing with cup and paste did not result in a s*R*_a_ reduction (Table 1). The final cleaning with the sonic toothbrush did not lead to any further change in the s*R*_a_ value in the test group (Figure 3). In the control group, neither polishing with cup and paste nor the application of the sonic toothbrush caused a significant change in s*R*_a_ values (Figure 3). The absolute values are presented in Table S1.

**TABLE 1:** Changes in s*R*_a_ and s*R*_z_.

	Test group *n* = 27				Control group *n* = 27		Intergroup differences Δ Control‐test	
	Δ air‐polishing‐baseline Step I	Δ rubber cup‐air‐polishing Step II	Δ rubber cup‐baseline Step I + II	Δ sonic brush‐air‐polishing Step I–III	Δ rubber cup‐baseline Step II	Δ sonic brush‐rubber cup Step III	Δ rubber cup‐baseline Step II	Δ sonic brush‐baseline Step II + III
s*R*_a_	0.168* ± 0.143	−0.042 ± 0.082	−0.002 ± 0.054	−0.044 ± 0.081	−0.009 ± 0.075	−0.006 ± 0.052	−0.136^#^ ± 0.147	−0.140^#^ ± 0.139
s*R*_z_	−0.471 ± 4.857	−0.759 ± 3.818	0.208 ± 2.807	−0.551 ± 3.563	0.368 ± 3.241	−0.416 ± 2.564	1.598 ± 5.122	0.974 ± 4.501

*Note*: Mean differences (Δ) ± standard deviation for s*R*_a_ and s*R*_z_ (in μm) between different treatment steps of the test and control groups and intergroup differences. Statistically significant differences between treatment steps are indicated with * and those between groups are indicated with ^#^ (paired *t*‐test).

**FIGURE 4:**
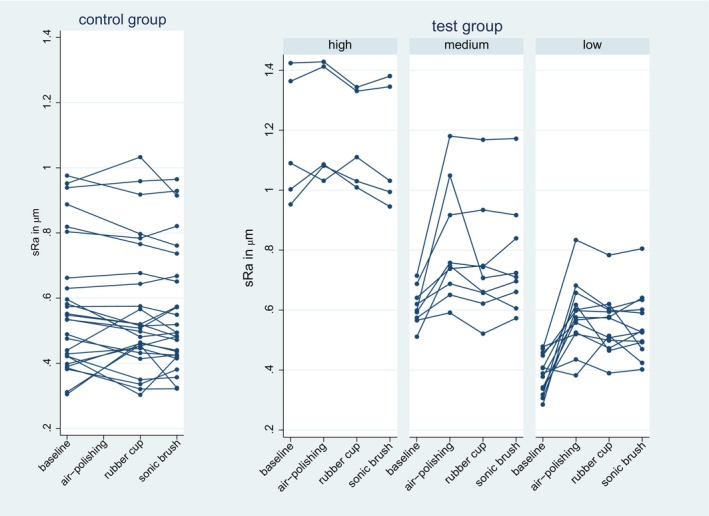
s*R*_a_ distribution. Mean s*R*_a_ values (in μm) for each participant for the test and the control group teeth at the baseline and after the various treatment steps.

The initial roughness level of the dentin surface influenced the direction of the surface roughness change. In the test group, s*R*_a_ values of aspects with low baseline values were more likely to be increased by air‐polishing than those that had high s*R*_a_ values at the baseline. No such effect was observed in the control group (Figure 4). A comparison of the test and control groups showed significantly larger s*R*_a_ changes in the test group, both after polishing with cup and paste (0.136 μm ± 0.147) and after cleaning with the sonic toothbrush (0.140 μm ± 0.139). These results can be attributed to the significant s*R*_a_ increase due to air‐polishing application.

#### 3.2.2 s*R*_z_

**FIGURE 5:**
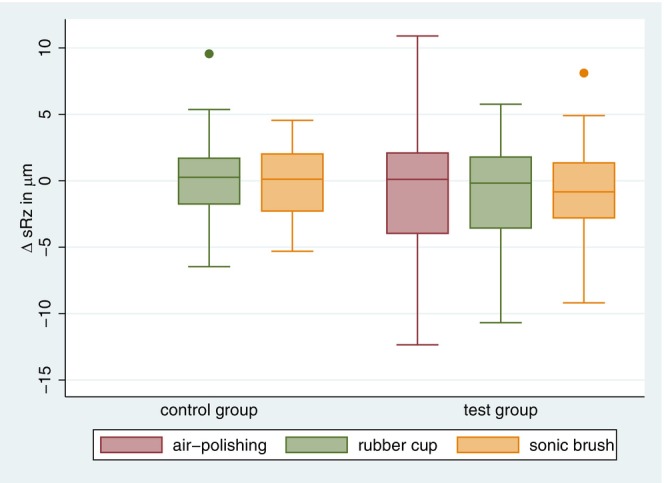
Differences in s*R*_z_. Boxplots showing differences in s*R*_z_ (in μm) between air‐polishing/baseline, air‐polishing/rubber cup and rubber cup/sonic toothbrush for the test group and between rubber cup/baseline and rubber cup/sonic toothbrush for the control group.

Regarding the s*R*_z_, air‐polishing application caused a slight but nonsignificant reduction in the average roughness depth in the test group compared to the baseline values (Table 1). Furthermore, polishing with cup and paste following air‐polishing did not result in a significant s*R*_z_ change, nor did subsequent treatment with the sonic toothbrush. In the control group, the s*R*_z_ value did not significantly change after polishing with cup and paste or after sonic toothbrushing (see Figure 5). When comparing the two groups, there was no statistically significant difference in the s*R*_z_ change in relation to the initial values after polishing with cup and paste. There was also no statistically significant difference between the two groups after cleaning with the sonic toothbrush.

### 3.3 Three‐dimensional visualization of roughness data

**FIGURE 6:**
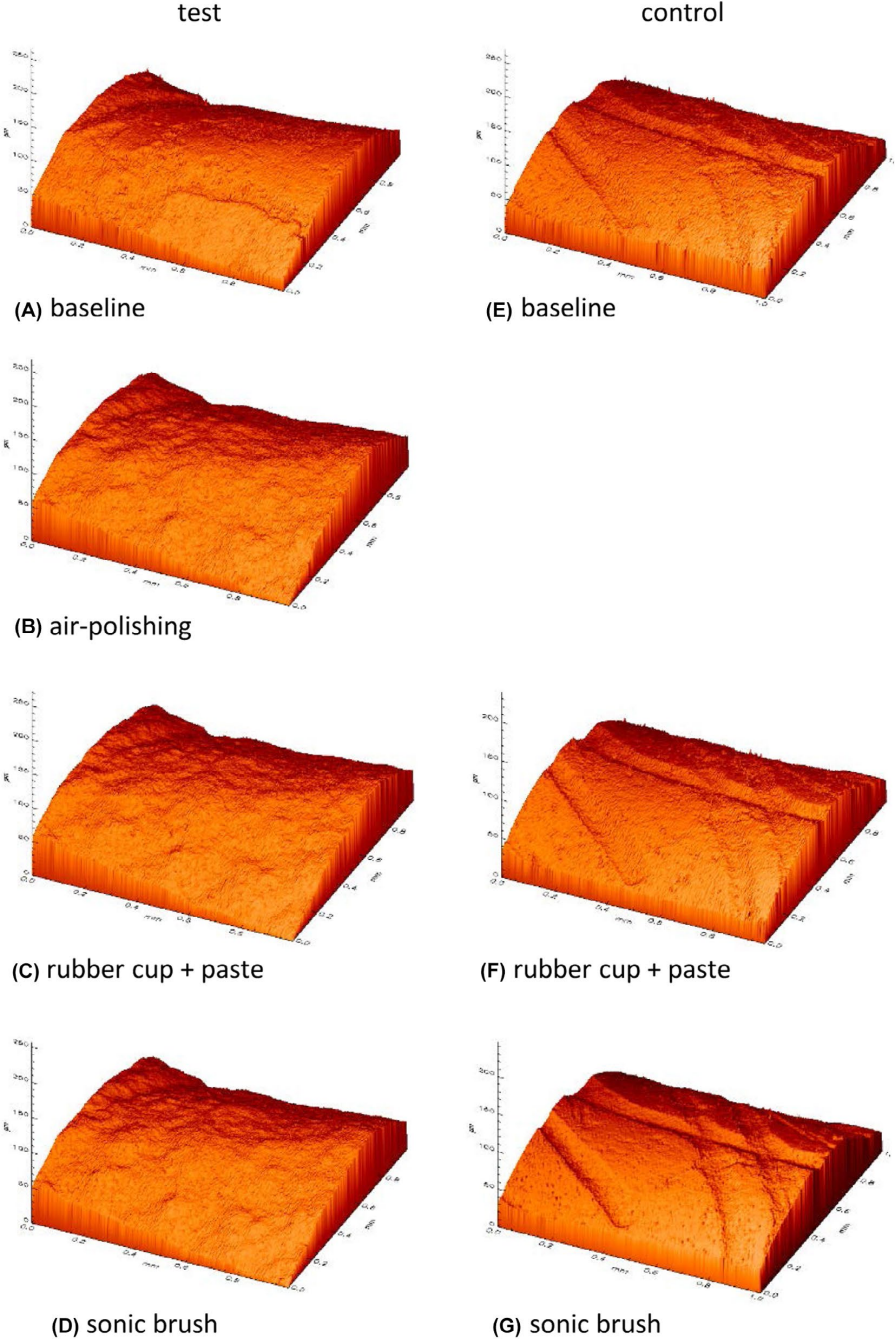
3D visualization of the profilometrical data. Depiction of surface roughness from two representative teeth at the baseline and after the air‐polishing, rubber cup and sonic toothbrush treatment steps in the test group (A–D) and at baseline and after the rubber cup and sonic toothbrush steps in the control group (E–G).

3D visualizations of individual representative root dentin surfaces from the test and control groups were compiled to additionally assess changes in the surface topography (Figure 6A–G). These images serve as an additional visual representation of the previously described results. In the test tooth, the root surface after air‐polishing application (b) appeared considerably more undefined and uneven compared to the initial situation (a). Further structural changes of the surfaces after the subsequent treatment steps with polishing cup and paste (c) and the sonic toothbrush (d) were not detected. In the control tooth, the traces of the curette that broke up the dentin surface when removing the biofilm at the beginning of the study session were clearly visible (e). After application of the polishing cup and paste (f) and the sonic toothbrush (g), no changes in the surface texture were detected.

## 4 DISCUSSION

The findings of this randomized clinical trial suggested that air‐polishing with erythritol slightly increases the roughness of dentin surfaces. A subsequent polishing step using a rubber cup and polishing paste did not significantly lower the surface roughness (s*R*_a_). Furthermore, a final removal of polishing paste residue using a sonic toothbrush did not lead to a change in roughness, so it cannot be assumed that possible cavities were obscured by the paste.

The mean roughness increase of 0.17 μm after erythritol seems to be rather low because sodium bicarbonate has been shown to increase the dentin roughness by up to 4.17 μm.^5^ Nevertheless, it is unclear whether minor roughening could lead to faster adhesion of extrinsic stains^22^, ^23^ or promote biofilm formation. An investigation of different dental materials found an apparent threshold of approximately 0.2 μm above which biofilm formation is significantly increased.^24^, ^25^ However, it is unclear whether these results can be generalized to dentin.

The results of this clinical study confirm the findings from the previous ex‐vivo study mentioned earlier with an almost identical study protocol as s*R*_a_ increased by 0.16 μm after air polishing used on natural dentin samples.^17^ Due to the heterogeneity of investigation approaches in the few existing in vitro studies, it is difficult to compare our results to other previous findings. In particular, there is little data about the effect of using low‐abrasive powders with air polishing on dentin surfaces. Kröger et al.^13^ found a maximum *R*_a_ value of 0.30 μm after constant air‐polishing with erythritol on dentin surfaces without any movement. Because more detailed information is not given in the publication and constant spraying is not suitable in the clinical setting, it is difficult to compare our results with those of that study. Nevertheless, 0.30 μm is not markedly different from the s*R*_a_ values found in the present study. Sultan et al.^14^ investigated the surface roughness of cementum after the use of glycine powder with a particle size of 45–60 μm; however, no treatment duration was given. A mean *R*_a_ value of 0.5 μm was measured for the root cementum surface, but in addition to the lack of absolute values, no information was provided regarding the changes caused by the treatment. Nevertheless, this value is in the range of our measured absolute s*R*_a_ values after the use of erythritol on dentin, even though many study parameters did not match. Arefnia et al.^12^ investigated the effect of air polishing on the surface roughness of enamel and cementum using erythritol powder with a particle size of 7 μm. Only absolute values are presented in the report, which showed higher *R*_a_ and *R*_z_ values of cementum surfaces after treatment with air‐polishing alone than after air‐polishing with subsequent rubber cup polishing. These results are different from our findings, likely because of the different substrate (cementum vs. dentin) and the different diameter of the powder particles. In addition, polishing pastes with descending RDA values of 170 > 120 > 40 > 7 were used in sequence for the cup and paste polishing. This might explain the decrease in roughness after this step. Because only one paste (RDA 40) was used in the present work, the effect of such a descending series cannot be assessed and should be considered in future studies.

From a clinical point of view, the pretreatment for biofilm removal has to be discussed. In order to create the same conditions for all participants, the biofilm on the tooth surface to be examined was stained and carefully removed with a curette. This procedure for sure does not correspond to the actual indication for the use of the curette within professional tooth cleaning. The study setting therefore differs from everyday clinical practice and may represent a limitation of the study as overlapping effects could possibly have arisen due to the pre‐treatment with the curette before the use of air polishing. In this study, however, it was not the absolute values that were compared, but the differences between the roughness values obtained from the impression directly before and after the air polishing. Therefore, the sole effect of air polishing could be shown by calculating the differences. Furthermore, in a previous study, in which the individual effects of curette, air polishing and polishing with cup and paste were examined independently^17^ as well as the interactions thereof, a similar increase in roughness was shown for s*R*_a_, which corresponds to the results from the calculated differences in this clinical study. In our study, the test group tended to show decreases in roughness when the initial roughness values were high. Conversely, when the initial roughness was low, the roughness tended to increase more after air‐polishing. Additionally, in the 3D visualization, air‐polishing resulted in a rather homogeneous roughening of an entire area compared to, for example, the use of curettes, whose application tends to leave grooves or craters. These two findings suggest that air‐polishing is more likely to level off larger, more severe irregularities, especially on dentin surfaces that have previously been treated with ultrasonic instruments or other treatments that significantly increase surface roughness. Furthermore, a certain performance bias cannot be completely ruled out due to a single operator in this study. The generalisability of the results could therefore be limited, but the same results can certainly be achieved by experienced users/operators. Even if there are the mentioned limitations, this is the first study investigating the effect of surface polishing procedures on roughness values under clinical conditions. The replica technique made it possible to perform the treatment steps as part of a clinical study while simultaneously measuring the dentin surfaces with high‐precision confocal optical profilometry. Regarding the surface parameters, most previous in vitro studies measuring the roughness of dentin surfaces have used individual linear profiles to calculate the *R*_a_ and *R*_z_ values. In the present study, a representative area of 1 by 1 mm was measured and surface‐related roughness values (s*R*_a_ and s*R*_z_) were calculated. This method increased the reliability of the data and therefore reduced the risk of bias. Nevertheless, based on the present results of a single‐visit investigation of individual polishing steps, it is difficult to draw conclusions about the effect of repeated applications on the roughness of dentin surfaces. Therefore, future studies should investigate how repeated treatments with air‐polishing and/or polishing cups and paste affect dentin surfaces in terms of roughness, substance loss, biofilm formation, and the adhesion of extrinsic stains. These questions are highly relevant, as professional mechanical biofilm removal is carried out daily in dental offices worldwide and has been for many years, especially for periodontitis patients. Therefore, more comparable in vitro studies and additional randomized clinical trials are needed.

## 5 CONCLUSION

Air‐polishing with erythritol powder led to a slight increase in the arithmetic average roughness of dentin surfaces. A subsequent additional polishing step with a rubber cup and paste did not lead to a change in this parameter, and therefore it does not appear to be necessary for surface smoothening. Furthermore, polishing paste does not seem to mask surface roughness by concealing the surface morphology.

## 6 CLINICAL RELEVANCE

### 6.1 Scientific rationale for the study

The effects of air‐polishing on exposed root dentin surfaces are largely unknown, as there are only a few studies, and they show heterogeneous results.

### 6.2 Principal findings

Air‐polishing with erythritol resulted in a slight increase in the arithmetic average surface roughness (s*R*_a_), and subsequent polishing with a rubber cup and paste did not lead to a change in this parameter. The maximum peak‐to‐valley height (s*R*_z_) did not change for either treatment step.

### 6.3 Practical implications

An additional polishing step with a rubber cup and paste is not necessary after the use of air‐polishing with erythritol powder on exposed dentin surfaces.

## AUTHOR CONTRIBUTIONS

P.R.‐K., N.S. and A.B.K. conceived the ideas; A.‐S.B. collected the data; K.V., N.S. and A.B.K. analysed the data; and A.B.K. led the writing. All authors read and approved the final version of the manuscript.

## FUNDING INFORMATION

This study was fully funded by the Association for Dental Infection Control (ADIC).

## CONFLICT OF INTEREST STATEMENT

All authors declare that they have no conflicts of interest.

## ETHICS STATEMENT

The study protocol was approved by the Ethics Committee of the University Medical Center Freiburg with a positive vote granted on 3/12/2020 (EK No. 625/19).

## PATIENT CONSENT STATEMENT

All enrolled participants gave their written informed consent for study participation and signed a data privacy statement.

## CLINICAL TRIAL REGISTRATION

Before the start of recruitment, the study was registered in an international trial register (German Clinical Trial Register number DRKS00021624, registration date 5/11/2020, https://drks.de/search/de/trial/DRKS00021624).

## Supporting information


Table S1

## Data Availability

The data that support the findings of this study are available from the corresponding author upon reasonable request.

## References

[1] Papapanou PN , Sanz M , Buduneli N , et al. Periodontitis: consensus report of workgroup 2 of the 2017 world workshop on the classification of periodontal and peri‐implant diseases and conditions. J Periodontol. 2018;89(Suppl 1):S173‐S182. doi:10.1002/JPER.17-0721 29926951

[2] Ramseier CA , Woelber JP , Kitzmann J , Detzen L , Carra MC , Bouchard P . Impact of risk factor control interventions for smoking cessation and promotion of healthy lifestyles in patients with periodontitis: a systematic review. J Clin Periodontol. 2020;47:90‐106. doi:10.1111/jcpe.13240 31912512

[3] Berkstein S , Reiff RL , McKinney JF , Killoy WJ . Supragingival root surface removal during maintenance procedures utilizing an air‐powder abrasive system or hand scaling. An in vitro study. J Periodontol. 1987;58(5):327‐330. doi:10.1902/jop.1987.58.5.327 3295185

[4] Petersilka GJ , Bell M , Mehl A , Hickel R , Flemmig TF . Root defects following air polishing. J Clin Periodontol. 2003;30(2):165‐170. doi:10.1034/j.1600-051x.2003.300204.x 12622860

[5] Bühler J , Amato M , Weiger R , Walter C . A systematic review on the effects of air polishing devices on oral tissues. Int J Dent Hyg. 2016;14(1):15‐28. doi:10.1111/idh.12120 25690301

[6] Petersilka GJ , Tunkel J , Barakos K , Heinecke A , Häberlein I , Flemmig TF . Subgingival plaque removal at interdental sites using a low‐abrasive air polishing powder. J Periodontol. 2003;74(3):307‐311. doi:10.1902/jop.2003.74.3.307 12710749

[7] Petersilka SD , Häberlein I , Heinecke A , Flemmig TF . Subgingival plaque removal in buccal and lingual sites using a novel low abrasive air‐polishing powder. J Clin Periodontol. 2003;30(4):328‐333. 12694431 10.1034/j.1600-051x.2003.00290.x

[8] Wennström JL , Dahlén G , Ramberg P . Subgingival debridement of periodontal pockets by air polishing in comparison with ultrasonic instrumentation during maintenance therapy. J Clin Periodontol. 2011;38(9):820‐827. doi:10.1111/j.1600-051X.2011.01751.x 21736599

[9] Petersilka G , Heckel R , Koch R , Ehmke B , Arweiler N . Evaluation of an ex vivo porcine model to investigate the effect of low abrasive airpolishing. Clin Oral Investig. 2018;22(7):2669‐2673. doi:10.1007/s00784-018-2536-5 PMC6097043 29959595

[10] Flemmig TF , Arushanov D , Daubert D , Rothen M , Mueller G , Leroux BG . Randomized controlled trial assessing efficacy and safety of glycine powder air polishing in moderate‐to‐deep periodontal pockets. J Periodontol. 2012;83(4):444‐452. doi:10.1902/jop.2011.110367 21861637

[11] Petersilka GJ . Subgingival air‐polishing in the treatment of periodontal biofilm infections. Periodontol. 2011;55(1):124‐142. doi:10.1111/j.1600-0757.2010.00342.x 21134232

[12] Arefnia B , Koller M , Wimmer G , Lussi A , Haas M . In vitro study of surface changes induced on enamel and cementum by different scaling and polishing techniques. Oral Health Prev Dent. 2021;19(1):85‐92. doi:10.3290/j.ohpd.b927695 33511822 PMC11640971

[13] Kröger JC , Haribyan M , Nergiz I , Schmage P . Air polishing with erythritol powder—in vitro effects on dentin loss. J Indian Soc Periodontol. 2020;24(5):433‐440. doi:10.4103/jisp.jisp_414_19 33144771 PMC7592611

[14] Sultan F , Joshi NV , Rathod VJ . In vitro analysis of surface roughness produced by an air polishing device and conventional root Planing on cementum: a profilometric study. J Indian Soc Periodontol. 2022;26(2):110‐116. doi:10.4103/jisp.jisp_594_20 35321304 PMC8936017

[15] Petersilka GJ , Bell M , Häberlein I , Mehl A , Hickel R , Flemmig TF . In vitro evaluation of novel low abrasive air polishing powders. J Clin Periodontol. 2003;30(1):9‐13. 12702105 10.1034/j.1600-051x.2003.300102.x

[16] Camboni S , Donnet M . Tooth surface comparison after air polishing and rubber cup: a scanning electron microscopy study. J Clin Dent. 2016;27(1):13‐18. 28390211

[17] Kruse AB , Fortmeier S , Vach K , Hellwig E , Ratka‐Krüger P , Schlueter N . Impact of air‐polishing using erythritol on surface roughness and substance loss in dental hard tissue: an ex vivo study. PLoS One. 2024;19(2):e0286672. doi:10.1371/journal.pone.0286672 38408064 PMC10896509

[18] World Medical Association . World medical association declaration of Helsinki: ethical principles for medical research involving human subjects. JAMA. 2013;310(20):2191‐2194. doi:10.1001/jama.2013.281053 24141714

[19] Schulz KF , Altman DG , Moher D , CONSORT Group . CONSORT 2010 statement: updated guidelines for reporting parallel group randomised trials. Int J Surg. 2011;9(8):672‐677. doi:10.1016/j.ijsu.2011.09.004 22019563

[20] Schlueter N , Ganss C , De Sanctis S , Klimek J . Evaluation of a profilometrical method for monitoring erosive tooth wear. Eur J Oral Sci. 2005;113(6):505‐511. doi:10.1111/j.1600-0722.2005.00253.x 16324141

[21] Tocha J . Die In‐vitro‐Auswirkungen konventioneller und experimenteller Pulverstrahlapplikationen auf Komposit‐, Dentin‐ und Schmelzoberflächen. GEB‐IDN/10265. Accessed November 8, 2022. http://geb.uni‐giessen.de/geb/volltexte/2013/10265/

[22] Watts A , Addy M . Tooth discolouration and staining: a review of the literature. Br Dent J. 2001;190(6):309‐316. doi:10.1038/sj.bdj.4800959 11325156

[23] Vieira‐Junior WF , Vieira I , Ambrosano GMB , Aguiar FHB , Lima DANL . Correlation between alteration of enamel roughness and tooth color. J Clin Exp Dent. 2018;10(8):e815‐e820. doi:10.4317/jced.54881 30305882 PMC6174023

[24] Bollen CM , Lambrechts P , Quirynen M . Comparison of surface roughness of oral hard materials to the threshold surface roughness for bacterial plaque retention: a review of the literature. Dent Mater. 1997;13(4):258‐269. doi:10.1016/s0109-5641(97)80038-3 11696906

[25] Teughels W , Van Assche N , Sliepen I , Quirynen M . Effect of material characteristics and/or surface topography on biofilm development. Clin Oral Implants Res. 2006;17(Suppl 2):68‐81. doi:10.1111/j.1600-0501.2006.01353.x 16968383

