# Supplementary material for: Impact of air‐polishing with erythritol on exposed root dentin: A randomized clinical trial
Source: Int J Dent Hyg. 2024 Jun 9;23(1):63–72. doi: 10.1111/idh.12835 (PMC11717964; doi:10.1111/idh.12835)
Supplement: Supplementary file 1 — Table S1 [file IDH-23-63-s001.docx]

**Supporting Material**

**Table S1 Absolute values of sRa and sRz (in µm)**

|  | | Test Group n=27 | | | | Control Group n=27 | | |
| --- | --- | --- | --- | --- | --- | --- | --- | --- |
|  |  | baseline | air-polishing | rubber cup | sonic brush | baseline | rubber cup | sonic brush |
| sRa | median | 0.511 | 0.682 | 0.622 | 0.641 | 0.535 | 0.508 | 0.494 |
|  | mean | 0.606 | 0.774 | 0.733 | 0.731 | 0.572 | 0.563 | 0.557 |
|  | sd | 0.308 | 0.281 | 0.268 | 0.267 | 0.200 | 0.197 | 0.187 |
|  | min | 0.285 | 0.382 | 0.390 | 0.402 | 0.305 | 0.303 | 0.322 |
|  | max | 1.424 | 1.428 | 1.343 | 1.380 | 0.976 | 1.033 | 0.965 |
| sRz | median | 12.076 | 10.276 | 8.866 | 9.516 | 11.314 | 11.219 | 10.322 |
|  | mean | 11.965 | 11.494 | 10.735 | 10.943 | 10.987 | 11.354 | 10.939 |
|  | sd | 3.925 | 4.773 | 3.970 | 4.102 | 3.664 | 3.366 | 3.391 |
|  | min | 5.614 | 6.953 | 6.755 | 6.728 | 4.472 | 6.228 | 6.489 |
|  | max | 22.581 | 24.489 | 19.714 | 22.468 | 24.932 | 18.465 | 19.628 |
